# Emergence of Ceftriaxone-Resistant *Neisseria gonorrhoeae penA*-60–Carrying Strains, Thailand, 2025

**DOI:** 10.3201/eid3206.251860

**Published:** 2026-06

**Authors:** Rossaphorn Kittiyaowamarn, Pongsathorn Sangprasert, Natnaree Girdthep, Sirintra Pharanut, Tawan Nongpian, Thanakorn Arunngamwong, Ruechakorn Kunkhajornphan, Ismael Maatouk, Magnus Unemo

**Affiliations:** Bangrak STIs Center, Department of Disease Control, Ministry of Public Health, Bangkok, Thailand (R. Kittiyaowamarn, P. Sangprasert, N. Girdthep, S. Pharanut, T. Nongpian); Bhattamakun Hospital, Chonburi, Thailand (T. Arunngamwong); Patong Hospital, Phuket, Thailand (R. Kunkhajornphan); World Health Organization, Geneva, Switzerland (I. Maatouk); Örebro University, Örebro, Sweden (M. Unemo); University College London Institute for Global Health, London, UK (M. Unemo)

**Keywords:** gonorrhea, bacteria, antimicrobial resistance, sexually transmitted infections, bacterial infections, Neisseria gonorrhoeae, ceftriaxone resistance, public health, Thailand

## Abstract

We report 5 gonorrhea cases caused by ceftriaxone-resistant *penA*–60-carrying *Neisseria gonorrhoeae* bacteria during January–April 2025 in Thailand, successfully cured by ceftriaxone therapy. Most patients had prior over-the-counter antimicrobial drug exposure. Ceftriaxone-resistant gonococcal strains are spreading in Thailand, and strengthened gonorrhea and resistance surveillance and effective antimicrobial stewardship programs are needed.

Antimicrobial-resistant *Neisseria gonorrhoeae* is a major global public health concern and has been designated as an urgent threat by the US Centers for Disease Control and Prevention (*1*). Ceftriaxone is the only remaining option for effective therapy of gonorrhea in most countries (*2*,*3*). In 2018, a gonococcal strain with resistance to ceftriaxone and high-level resistance to azithromycin was reported in England, and this extensively drug-resistant strain infected a patient in Thailand (*4*). Occasional ceftriaxone-resistant gonococcal strains with links to Thailand subsequently have been detected in several countries in Europe (*3*,*5*). However, the National Gonococcal Antimicrobial Resistance program in Thailand, implemented in 2003, did not identify any ceftriaxone-resistant strains until 2023, when 2 ceftriaxone-resistant isolates caused by the mosaic *penA*-60 allele were confirmed (*6*). In response, a previously described *penA*-60–specific PCR (*7*) was implemented for further investigation of ceftriaxone-resistant isolates. No ceftriaxone-resistant gonococcal isolates were found in 2024 in Thailand; however, during January–April 2025, national surveillance identified 5 ceftriaxone-resistant gonococcal isolates.

The National Gonococcal Antimicrobial Resistance program has been included in Thailand’s national surveillance since 2003, whereas the World Health Organization Enhanced Gonococcal Antimicrobial Surveillance Program (EGASP) was established in sentinel clinics in 2015 (*8*,*9*). Gonococcal isolates and patient data from the national surveillance system are collected from the Bangrak Sexually Transmitted Infections (STIs) Center in Bangkok and from 6 regional Offices of Disease Prevention and Control that cover the main regions of Thailand (Figure 1). Gonococcal isolates are obtained from symptomatic and asymptomatic male and female patients attending STI clinics across Thailand. The 5 EGASP sites (2 in Bangkok, 1 in Chiang Mai, and 2 in Pattaya) strengthen surveillance among men with urethritis and men who have sex with men (*8*,*9*). The local laboratories send all isolates with resistance to ceftriaxone, cefixime, or azithromycin to the Bangrak STIs Center for further confirmation of *N. gonorrhoeae* bacteria and antimicrobial MIC testing. We determined MICs of ceftriaxone, cefixime, azithromycin, gentamicin, and tetracycline by using Etest (bioMérieux, https://www.biomerieux.com) according to the manufacturer’s instructions. We further examined ceftriaxone-resistant isolates through a *penA*-60–specific real-time reverse transcription PCR (*7*) by using a CFX96 Real-Time PCR system (Bio-Rad Laboratories, https://www.bio-rad.com).

**Figure 1:**
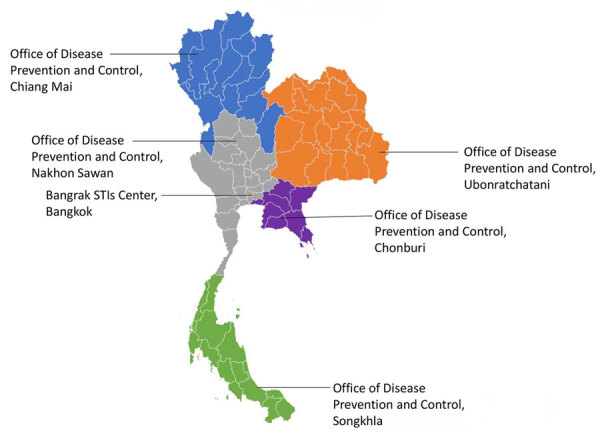
Locations of Bangrak STIs Center (Bangkok) and 6 regional Offices of Disease Prevention and Control that collect gonococcal isolates in national surveillance system (National Gonococcal Antimicrobial Resistance program and World Health Organization Enhanced Gonococcal Antimicrobial Surveillance Program), Thailand. STIs, sexually transmitted diseases.

## The Study

During January–April 2025, we identified 5 ceftriaxone-resistant gonococcal isolates among 272 isolates collected through the National Gonococcal Antimicrobial Resistance program in Thailand. The 5 isolates had been obtained from 5 male patients, 16–55 years of age, who sought care for dysuria at STI clinics in Thailand’s eastern region (4 patients in Pattaya, Chonburi Province) and southern region (1 patient in Phuket Province) of Thailand. Our case investigations were conducted by authorized public health officers according to the Communicable Disease Act in Thailand. Case identification information was confidential.

**Figure 2:**
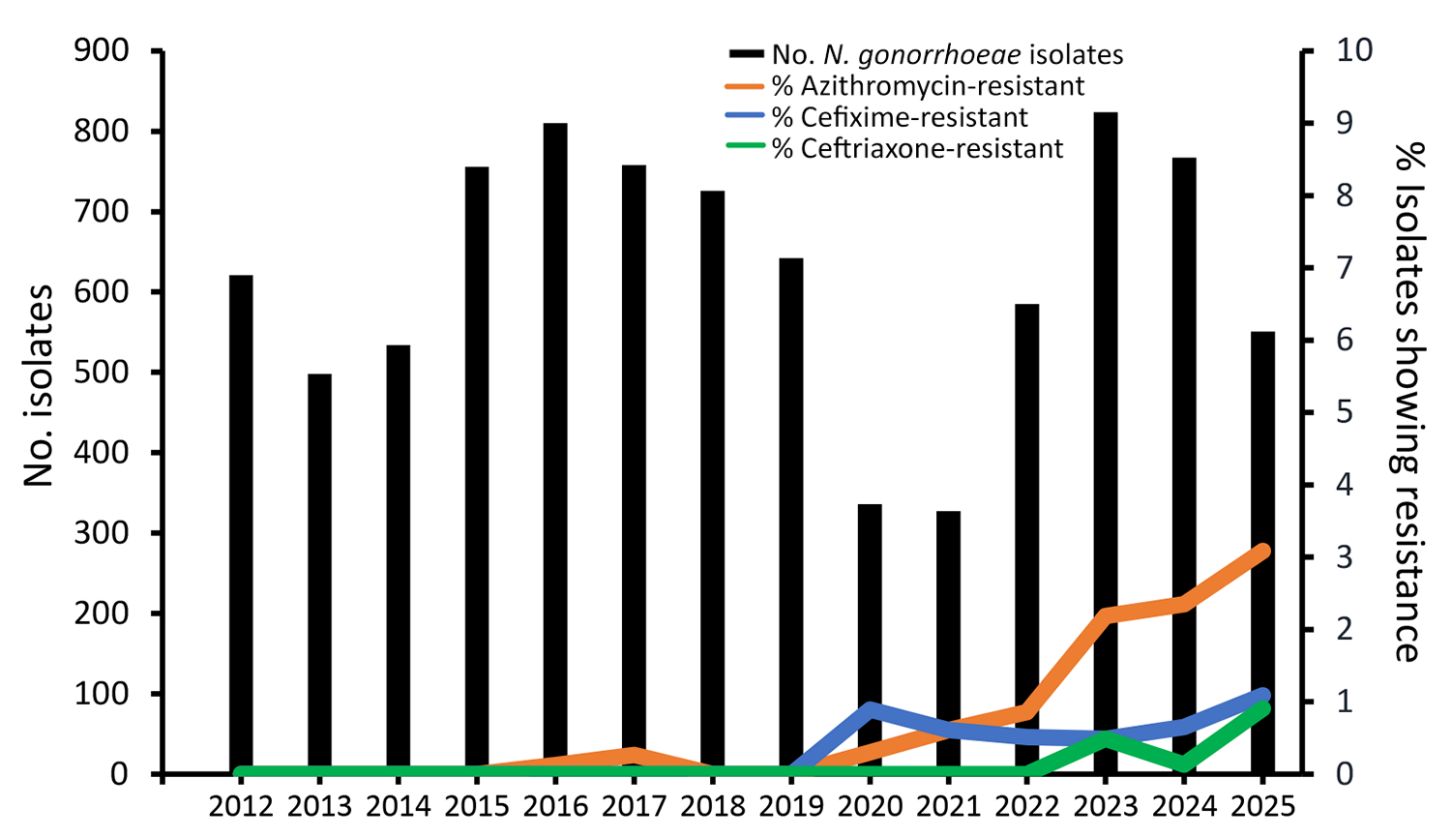
Number of *Neisseria gonorrhoeae* bacteria isolates examined and percentages of isolates with resistance to azithromycin, cefixime, and ceftriaxone in national surveillance system (National Gonococcal Antimicrobial Resistance program and World Health Organization Enhanced Gonococcal Antimicrobial Surveillance Program), by year, Thailand, January 2012–August 15, 2025. Isolates were identified by using Etest (bioMérieux, https://www.biomerieux.com).

The patients were from Thailand (n = 3), Russia (n = 1), and China (n = 1) (Table 1). All 5 patients were men who have sex with women, and 4 of them reported recent sexual contact with a casual partner, including a 1-night-stand partner and a female sex worker. Four patients reported self-treatment with oral antimicrobial drugs (mostly azithromycin 1 g) obtained from pharmacies in Pattaya Province before any visit to an STI clinic; however, because of persisting symptoms, they all subsequently visited an STI clinic. Gram-stained microscopic examination of urethral discharge from all 5 patients revealed gram-negative intracellular diplococci, and culture on modified Thayer-Martin medium confirmed the presence of gonococci. On the basis of European Committee on Antimicrobial Susceptibility Testing resistance breakpoints (*10*), we determined that all 5 gonococcal isolates were resistant to ceftriaxone (MIC >0.125 μg/mL) and cefixime (MIC >0.125 μg/mL), and 3 isolates showed high-level resistance to azithromycin (Table 1). Three of the 5 isolates also were nonsusceptible to ceftriaxone based on the Clinical Laboratory and Standards Institute clinical breakpoint (MIC >0.5 μg/mL) (*11*) (Table 2; Figure 2). All 5 isolates contained the *penA*-60 allele, which is associated with ceftriaxone resistance in *N. gonorrhoeae* bacteria (*2*–*7*,*12*,*13*). 

**Table 1:** Characteristics of case-patients with gonorrhea caused by ceftriaxone-resistant *Neisseria gonorrhoeae* isolates, Thailand, January–April 2025*

Case no.	Date	Nationality/ notified province (city)	Age, y	Occupation	Previous antimicrobial drug exposure	Symptoms	Travel history in past month	Prior sexually transmitted infection	No. partners in past 3 months
1	2025 Jan	Thai/ Chonburi (Pattaya)	35	Waiter	Yes (azithromycin 1 g twice)	Urethral discharge, dysuria	No	No	1 regular partner, 2 casual partners (FSWs)
2	2025 Feb	Thai/ Chonburi (Pattaya)	16	Waiter	Yes (azithromycin 1 g twice)	Urethral discharge, dysuria	No	No	1 regular partner living in different location, 1 one-night-stand partner
3	2025 Mar	Russian/ Phuket	55	–	–	Urethral discharge, dysuria	–	–	–
4	2025 Mar	Thai/ Chonburi (Pattaya)	23	Government official	Yes (quinolone and then azithromycin 1 g)	Urethral discharge, dysuria	No	Gonorrhea 10 y ago	1 casual partner (FSW)
5	2025 Apr	Chinese/ Chonburi (Pattaya)	25	–	Yes (unknown antibiotic)	Urethral discharge, dysuria	–	–	1 casual partner

*FSW, female sex worker; –, no data available.

**Table 2:** Diagnostic testing, treatment, and antimicrobial susceptibility of ceftriaxone-resistant *Neisseria gonorrhoeae* isolates, Thailand, January–April 2025*

Case no.	Syphilis rapid test	HIV testing	NAAT testing†	Treatment	MIC, µg/mL (R/S)‡	TOC, culture and NAAT†	Sexual partner testing
1	Negative	Negative	Pooled specimen from urine and OP site: positive	IM ceftriaxone 1 g + oral azithromycin 1 g	CRO 0.25 (R); CFM 1 (R); AZM 256 (R)	OP and urethral specimens: negative	Regular female partner (NG detected in pooled cervical and oropharyngeal specimens by Xpert CT/NG assay, but cervical and oropharyngeal cultures negative)
2	Negative	Negative	Not tested	IM ceftriaxone 1 g + oral doxycycline (200 mg/day for 7 d)	CRO 0.5 (R); CFM 2 (R); AZM 256 (R)	OP and urethral specimens: negative	One-night-stand partner (contact tracing not possible)
3	Not tested	Not tested	Pooled specimen from urine and OP site: positive	IM ceftriaxone 1 g	CRO 0.25 (R); CFM 2 (R); AZM 256 (R)	Not conducted	No partner reported
4	Negative	Negative	Not tested	IM ceftriaxone 1 g + oral doxycycline (200 mg/d for 7 d)	CRO 0.5 (R); CFM 1 (R); AZM 1 (S)	OP and urethral specimens: negative	FSW (contact tracing not possible)
5	Not tested	Not tested	Not tested	IM ceftriaxone 1 g + oral doxycycline (200 mg/d for 7 d)	CRO 0.5 (R); CFM 2 (R); AZM 0.125 (S)	Not conducted	Casual female partner (contact tracing not possible)

*AZM, azithromycin; CFM, cefixime; CRO, ceftriaxone; IM, intramuscular; NAAT, nucleic acid amplification test; NG, *Neisseria gonorrhoeae*; OP, oropharyngeal; R, resistant; S, susceptible; TOC, test-of-cure. †NAAT testing using Xpert CT/NG assay (Cepheid, https://www.cepheid.com). ‡Based on European Committee on Antimicrobial Susceptibility testing breakpoints (*10*).

On the same day results of the gram-stained microscopic examination of urethral discharge were available, all 5 patients were treated with intramuscular ceftriaxone (1 g) (n = 1) or intramuscular ceftriaxone (1 g) plus oral azithromycin (1 g) or a 7-day regimen of oral doxycycline (n = 4) in accordance with current gonorrhea management guidelines in Thailand (*14*). All 3 Thailand patients returned for test-of-cure 1–2 weeks after treatment; results of culture and the Xpert CT/NG assay (Cepheid, https://www.cepheid.com) from urethral and oropharyngeal sites were negative, and all 5 patients were asymptomatic. The 2 patients of foreign nationality did not return for test-of-cure. Only the first Thailand case-patient, who had a regular female partner, could bring his sexual partner to the STI clinic for examination and treatment. An Xpert CT/NG assay of pooled cervical and oropharyngeal specimens detected *N. gonorrhoeae* bacteria, whereas cervical and oropharyngeal cultures were negative. The woman was treated with intramuscular ceftriaxone (1 g) but did not return for test-of-cure.

## Conclusions

Increasing clinicians’ awareness of ceftriaxone-resistance is essential. Ceftriaxone is the last remaining gonorrhea treatment option and has acquired high levels of resistance in several countries in Asia, especially Cambodia, China, and Vietnam (*2*,*3*,*9*,*12*,*13*). Data from the National Gonococcal Antimicrobial Resistance program and EGASP in Thailand for 2015–2023 have shown that antimicrobial-resistant *N. gonorrhoeae* also is an emerging major concern in Thailand, particularly resistance to oral antimicrobial drugs such as ciprofloxacin (which exhibits >90% resistance), azithromycin, and (since 2020) cefixime, all of which are commonly available without a prescription at pharmacies in Thailand. However, before 2025, only 2 ceftriaxone-resistant gonococcal isolates had been confirmed in national surveillance of gonococcal antimicrobial resistance (*6*,*8*,*9*). 

We report 5 ceftriaxone-resistant isolates detected during January–April 2025 in Thailand. Most of the patients with ceftriaxone-resistant gonorrhea were initially self-treating with antimicrobial drugs instead of seeking care at STI clinics, and their sexual encounters involved 1-night stand partners and female sex workers, behaviors that are associated with further rapid transmission of the ceftriaxone-resistant gonococcal strains and substantial health risks and can complicate sexual contact tracing efforts. In Thailand, STIs have also become 1 of the top 5 conditions for which persons seek treatment at pharmacies (*15*), and the resulting widespread use of oral antimicrobial drugs probably has contributed to the increase in gonococcal antimicrobial resistance. Of note, we detected 3 additional ceftriaxone-resistant isolates during October–December 2025, which are undergoing further investigation. 

One limitation of our study is the lack of whole-genome sequencing of all the ceftriaxone-resistant isolates. However, we plan to conduct whole-genome sequencing in 2026.

The emergence of ceftriaxone-resistant gonorrhea cases in Thailand poses a serious public health challenge, and treatment of all gonorrhea cases with ceftriaxone, consistent with the updated gonorrhea management guidelines in Thailand, is critical (*14*). The increasing practice of self-treatment by purchase of antimicrobial drugs without a prescription at pharmacies (*15*), often resulting in inappropriate and failing treatment and further driving antimicrobial resistance, is a major concern. 

The spread of ceftriaxone-resistant gonococcal strains in Thailand and other countries in Asia (*2*,*3*,*9*,*12*,*13*) underscores the urgent need to promote safer sex practices and ensure access to professional medical care (diagnostics, treatment, and follow-up of patients and sexual contacts). In a promising development, novel drugs such as zoliflodacin and gepotidacin have been approved by the US Food and Drug Administration (*2*,*3*). However, encouraging responsible antimicrobial use and stewardship remains critical to protect health. Among the cases we describe, the 2 foreign male patients with ceftriaxone-resistant gonorrhea, who were subsequently lost to follow-up, underscore major gaps in continuity of care and highlight the urgent need to strengthen global gonococcal antimicrobial resistance surveillance and enhance coordinated international efforts to address gonococcal antimicrobial resistance. 
